# Prevalence and Molecular Epidemiology of Human T-Lymphotropic Virus (HTLV) Infection in People Living With HIV/AIDS in the Pará State, Amazon Region of Brazil

**DOI:** 10.3389/fmicb.2020.572381

**Published:** 2020-10-22

**Authors:** Samira Peixoto Alencar, Marlinda de Carvalho Souza, Ricardo Roberto de Souza Fonseca, Cláudia Ribeiro Menezes, Vânia Nakauth Azevedo, Andre Luis Ribeiro Ribeiro, Sandra Souza Lima, Rogério Valois Laurentino, Maria dos Anjos de Abreu Pina Barbosa, Felipe Bonfim Freitas, Aldemir Branco Oliveira-Filho, Luiz Fernando Almeida Machado

**Affiliations:** ^1^Biology of Infectious and Parasitic Agents Post-Graduate Program, Federal University of Pará, Belém, Brazil; ^2^Virology Laboratory, Institute of Biological Sciences, Federal University of Pará, Belem, Brazil; ^3^Evandro Chagas Institute, Ministry of Health of Brazil, Ananindeua, Brazil; ^4^Reference Unit Specialized in Infectious and Parasitic Diseases, Belém, Brazil; ^5^Vulnerable Populations Research Group, Institute for Coastal Studies, Federal University of Pará, Bragança, Brazil

**Keywords:** epidemiology, HIV, HTLV, coinfection, Amazon region, molecular epidemiology

## Abstract

The human T-lymphotropic virus (HTLV) is part of the group of retroviruses that share similar routes of transmission to the human immunodeficiency virus (HIV). Coinfection of these viruses can affect the clinical course of both infections, and reports have shown a quicker progression to AIDS and the development of HIV-related opportunistic infections. The current study investigated the demographic characteristics, prevalence, and the subtypes of HTLV among people living with HIV/AIDS (PLWHA) in the State of Pará, Northern Brazil. Blood samples were obtained from patients who were attending a reference unit that provides medical assistance to HIV-infected individuals in the State of Pará, Brazil, during the period of May 2016 to June 2017. Plasma samples were screened by ELISA tests to detect antibodies anti-HTLV-1/2. DNA and viral types were identified by real-time polymerase chain reaction (qPCR). All samples with viral DNA were submitted to nested PCR and nucleotide sequencing. The overall coinfection rate was 1.4% (5/368), and all samples were from subtype HTLV-1a. No cases of HTLV-2 infection were detected. The prevalence of HTLV-1 was higher in females (80%), individuals between 31 and 50 years of age, heterosexual, unmarried, with low monthly income, with secondary educational level or higher, sporadic condom usage, limited number of sexual partners, and no history of sexually transmitted infections. All samples from HTLV-1-infected patients were identified as strains belonging to the subtype 1a (Cosmopolitan), subgroup A (Transcontinental). This study identified that the prevalence of HIV/HTLV coinfection has dropped from 8 to 1.3% in the current investigation. There was a shift of HTLV subtype from a predominance of HTLV-2 infection in the past to an actual exclusively HTLV-1a. There was no significant association between economic, sociodemographic, and behavioral characteristics in HIV/HTLV coinfection.

## Introduction

The human T-lymphotropic virus (HTLV) is part of a family of human retroviruses with four types, where the HTLV-1 and HTLV-2 are the most well-studied subtypes of HTLV. The HTLV-1 is associated with some severe diseases such as HTLV-1-associated myelopathy/tropical spastic paraparesis (HAM/TSP) and adult T-cell leukemia (ATL), whereas the HTLV-2 was not yet clearly associated with any human disorder (Gessain and Mahieux, 2012; Martinez et al., 2019). Coincidently, the route of transmission of the HTLVs is similar to another retrovirus, the human immunodeficiency virus (HIV) that is well-known worldwide to cause the acquired immunodeficiency syndrome (AIDS) (Gessain et al., 1986; Ticona et al., 2013). Transmission occurs mainly through vertical transmission (mother to child), unprotected sex, and exposure to infected blood such as blood transfusion and injectable drug users (Yoshida et al., 1982). These two retroviruses not only share transmission routes but also are found in coinfection, which can result in quicker progression to AIDS, poorer prognosis of HIV-related opportunistic infections, and a higher risk of developing neurological manifestations; however, the real impact of this coinfection remains controversial (Brites et al., 2009; Silva et al., 2012; de Mendoza et al., 2019).

The HTLV-1 is the most disseminated globally and is considered endemic in southern Japan, in some regions of Iran and Melanesia, sub-Saharan Africa, the Caribbean, and South America (Gessain and Cassar, 2012). Brazil shows a high prevalence of HTLV infection with high variance in different groups and regions (Romanelli et al., 2013). In the State of Pará, which is in the northern region of Brazil and characterized by large areas of the Amazon rainforest, limited infrastructure, and low human development, the prevalence varies from 0.1 to 1.4%, reaching a peak of 3% among people who used illicit drugs (PWUDs) (de Aguiar et al., 2017; Guerra et al., 2018; Silva et al., 2018; Oliveira-Filho et al., 2019). The prevalence of HTLV-2 is lower than HTLV-1 in most places; however, it particularly affects specific populations, such as native Amerindian groups and PWUDs (Ishak et al., 1995; Catalan-Soares et al., 2005; Murphy et al., 2015; Braço et al., 2019).

Molecular studies based on the long terminal repeat (LTR) region have classified HTLV-1 in seven genetic subtypes (1a–1g) and HTLV-2 in four subtypes (2a–2d) (Vandamme et al., 1998; Gessain and Cassar, 2012). The HTLV-1a or Cosmopolitan subtype is the most prevalent worldwide (Gessain and Cassar, 2012), as well as in Brazil (Castro et al., 2018; Ribeiro et al., 2018) and in Pará state (Vallinoto et al., 2006; Nobre et al., 2018). The prevalence of HTLV coinfecting people living with HIV/AIDS (PLWHA) varies according to the studied population and the geographical region. In Mozambique, the prevalence of coinfection in children was 3.9% (Manhiça et al., 2017), whereas in inmates, it was 1.5% (Caterino-de-Araujo et al., 2015). In Spain, the prevalence of coinfection was 3.2% (de Mendoza et al., 2019), 12% in Iran (Pirayeshfard et al., 2018), and 4.9% in Nigeria (Nasir et al., 2015). In Brazil, data show that HIV/HTLV coinfection varies from 0.8 to 6.4% (Morimoto et al., 2005; de Oliveira et al., 2012; Galetto et al., 2014; Caterino-de-Araujo et al., 2015; Kozlowski et al., 2016); most of them subtyped as the HTLV-1a.

In Brazil, in 2018, 43,941 new cases of HIV infection were diagnosed, being 5,084 cases (11.6%) in the north region of the country, where Pará was the state with the highest number of cases. Related to Brazil, the proportion of new HIV infection cases has remained stable over the last years (4.1% in 2016, 4.2% in 2017, and 4.2% in 2018) (Brasília, 2019). Some studies show that the prevalence of HIV infection in Pará may vary from 0.3% (Guerra et al., 2018) to 0.6% (Vallinoto et al., 2016).

Conversely, only two studies have investigated the prevalence of HTLV coinfecting PLWHA in the State of Pará, showing an overall prevalence of HTLV infection of 7.4% in 1998 (Vallinoto et al., 1998) and 3.5% in 2005 (Laurentino et al., 2005), with a predominance of HTLV-2 subtype with a rate of 1.74:1 and 2:1 over HTLV-1, respectively. The current study aimed to investigate the current prevalence of HIV/HTLV coinfection, circulating HTLV subtypes, and demographic characteristics in PLWHA in the State of Pará, Northern Brazil.

## Materials and Methods

### Type of Study and Ethical Aspects

The present study is descriptive, cross-sectional, and observational. The Human Research Ethics Committee of the Health Sciences Institute, Federal University of Pará, approved the study under number 2.601.161. The participants were informed about the study objectives and agreed to participate in the research; they signed a consent form and then answered an epidemiological questionnaire via a confidential structured interview face-to-face. The questionnaire contained questions regarding age, sex, years of education, marital status, and condom use.

### Ethics Statement

Written informed consent was obtained from all five coinfected HIV/HTLV individuals for the publication of any potentially identifiable images or data included in this article.

### Study Sample

A total of 368 PLWHA who underwent clinical and laboratorial care at the Specialized Reference Unit on Special Infectious and Parasitic Diseases (Unidade de Referência Especializada em Doenças Infecciosas e Parasitárias Especiais-UREDIPE) under the Executive Secretariat of Public Health of the State of Pará (Secretaria Executiva de Saùde Pùblica do Estado do Pará-SESPA) participated in the study from May 2016 to June 2017. The UREDIPE is a reference unit where the clinic–laboratorial attendance of the PLWHA from many of the 144 counties of the Pará state is performed. The subjects participating in the present study were from 63 cities of the Pará state.

The inclusion criteria were: ≥18 years old, confirmed HIV infection, agreement to participate in the study, and signing of the patient informed consent form. The exclusion criteria were patients with cognitive impairment who were unable to answer the questionnaire in an appropriate way.

### Serology

Peripheral blood (10 ml) was collected from each participant in a tube containing ethylenediaminetetraacetic acid (EDTA) as an anticoagulant. The samples were transported to the Virology Laboratory of the Biological Sciences Institute, Federal University of Pará. Plasma and formed elements were separated by centrifugation at 8,944 *g* for 15 min, transferred to cryotubes, and frozen at −20°C until serological and molecular analyses.

Plasma samples were screened for anti-HTLV-1/HTLV-2 antibodies using a qualitative enzyme-linked immunosorbent assay (ELISA; Murex HTLV I + II; DiaSorin, Saluggia, Italy) in the Virology Section of Evandro Chagas Institute/Health Surveillance Secretariat, Ministry of Health of Brazil. Reactive or inconclusive results were confirmed using Real-time PCR (Applied Biosystems, Foster City, CA, United States), which also differentiated between HTLV-1 and HTLV-2 infection.

### Real-Time PCR

DNA was extracted using PureLink^TM^ Genomic DNA Kit (Invitrogen, CA, United States) according to the manufacturer’s protocol to amplify *5′ LTR* and *pol* regions. The qPCR reactions were prepared using the TaqMan^®^ Universal master mix according to protocol: 15 μl of Master Mix, 10.5 μl of water, 1.5 μl of Assay-by-Design (primer and probe set), and 3 μl of DNA in a final volume of 30 μl. The cycling protocol used was one cycle of 50°C for 2 min and 95°C for 10 min and 50 cycles of 95°C for 15 s and 60°C for 1 min. An endogenous control (human albumin gene) and the non-homologous regions of the *pol* gene (186 bp) of HTLV-1 and HTLV-2 were used. The primers used were HTLV-1F 5′-GAACGCTCTAATGGCATTCTTAAAACC-3′), HTLV-1R (5′-GTGGTTGATTGTCCATAGGGCTAT-3′), HTLV-2F (5′-CAACCCCACCAGCTCAGG-3′), HTLV-2R (5′-GGGAAGGTTAGGACAGTCTAGTAGATA-3′), Albumin F (5′-GCTCAACTCCCTATTGCTATCACA-3′), and Albumin R (5′-GGGCATGACAGGTTTTGCAATATTA-3′) as previously described (Ribeiro et al., 2019). The probe sequences used were FAM-5′-ACAAACCCGACCTACCC-3′-NFQ (HTLV-1), FAM-5′-TCGAGAGAACCAATGGTATAAT-3′-NFB (HTLV-2), and FAM-5′-TTGTGGGTGTAATCAT-NFQ (Albumin) (Tamegão-Lopes et al., 2006).

### Nested PCR and Sequencing

The samples that were confirmed as positive for HTLV-1 were subjected to nested PCR reactions for amplification of the *5*′*LTR*. The first and second round PCR reactions were run in a final volume of 50 μl containing 400 ng of extracted DNA, 10 μM of each dNTP, 20 pmol/μl of each primer, 50 μM MgCl_2_, 1 × buffer (50 mM KCl, 10 mM Tris–HCl pH 8.3), and 5 units of Taq DNA polymerase. In each amplification reaction, after initial denaturation at 94°C for 5 min, 35 cycles were performed, with 40 s at 94°C, followed by 30 s at 57°C and 1 min at 72°C, followed by a final extension for 10 min at 72°C. The primers used for the first step of reaction were LTR-I.01 (5′-TGACAATGACCATGAGCCCCAA-3′) and LTR-I.02 (5′-CGCGGAATAGGGCTAGCGCT-3′), and for the second step, the primers used were LTR-I.03 (5′-GGCTTAGAGCCTCCCAGTGA-3′) and LTR-I.04 (5′-GCCTAGGGAATAAAGGGGCG-3′). The nested PCR products were visualized after electrophoresis (100 V/45 min) on 2% agarose gel in 1 × TAE buffer (50 × TAE stock solution-1.6 M TrisBase, 0.8 M sodium acetate, and 40 mM EDTA-Na2 in 1,000 ml of deionized water) that contained 5 μl of ethidium bromide (10 mg/ml) using a transilluminator with an ultraviolet light source.

Both strands of the *5*′*LTR* were sequenced three times in both directions using ABI Prism BigDye^TM^ Terminator Ready Reaction Cycle Sequencing kit, version 3.1 (Applied Biosystems, Foster City, CA, United States). After precipitation of the reaction product, the samples were denatured and sequenced in an automated ABI 3130 sequencer (Applied Biosystems) following the manufacturer’s protocol.

### Sequence and Phylogenetic Analysis

All sequences were edited and aligned using AliView software (Larsson, 2014). The subtypes were determined using phylogenetic analyses with HTLV reference sequences, obtained from the National Center for Biotechnology Information^1^. To verify the clustering of HTLV sequences, maximum-likelihood (ML) phylogenetic trees were reconstructed with the PhyML 3.1 (Guindon et al., 2010) under the best nucleotide substitution model, selected by the SMS (Smart Model Selection) software (Lefort et al., 2017) integrated into the PhyML Web server. The heuristic trees search was performed using the SPR branch-swapping algorithm, and the branch support was calculated with the approximate likelihood-ratio (aLRT) SH-like test. The tree was drawn with FigTree 1.4.4^2^. The sequences obtained in this study were deposited in GenBank (MT941570–MT941574).

### Statistical Procedures

Assuming the PLWHA population size of 10,000 individuals, margin of type I error of ±5%, confidence level of 95%, and sample proportion equal to 50%, the minimum sample size was calculated to be 370 participants. The sample number was calculated using the OpenEpi software^3^. Furthermore, all study data collected were entered into an Excel database and converted to BioEstat. To compare the epidemiological characteristics of the PLWHA in relation to the HIV/HTLV coinfected subjects, the Fisher’s exact test and the G test were used. The Fisher’s exact test and G test were used to compare the epidemiological characteristics of PLWHA in relation to those coinfected with HIV/HTLV. Odds ratio (OR) and associated 95% confidence interval (CI) were used as measures of the strength of independent association between HTLV/HIV infection and individual variables and the variation of this strength. The numbers of HTLV-positive and -negative cases detected in this study were also compared with the results found in two studies conducted with PLWHA in the Brazilian State of Pará in 1998 (Vallinoto et al., 1998) and 2005 (Laurentino et al., 2005) in order to assess the epidemiological trend of HTLV infections. A *p*-value <0.05 significance was considered for all analyses. All statistical procedures were conducted in BioEstat 5.0 for Windows.

## Results

This study accessed information and biological samples of 368 PLWHA treated at the main specialized service for HIV/AIDS in the Brazilian State of Pará. Although the study accessed many PLWHA attended in UREDIPE from May 2016 to June 2017, it was not enough to achieve the estimated minimum sample size (*n* = 370). However, the value is very close and can be considered safe and representative of the population, as predicted in the calculation.

**FIGURE 1:**
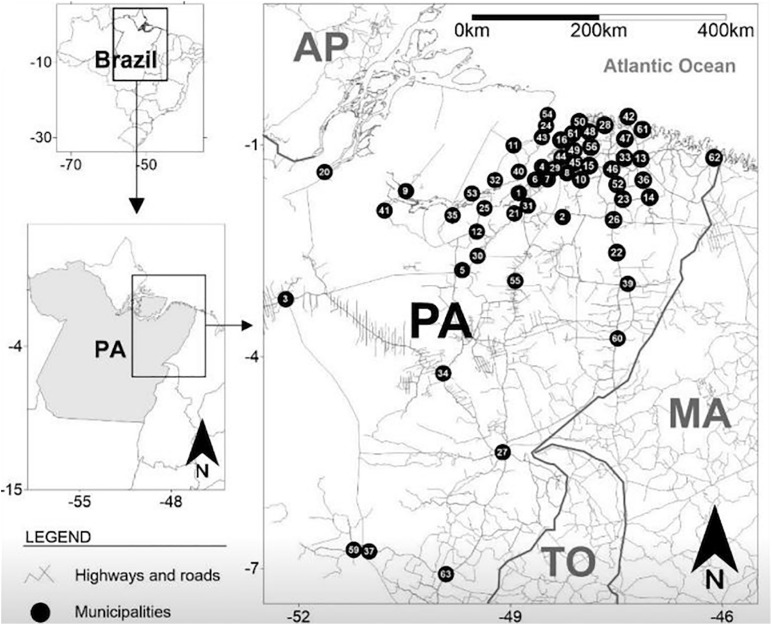
Geographical distribution of cities with participants enrolled in this study and living with HIV/AIDS in the State of Pará, Brazil. Black circles represent the locations of the cities on the state map, and numbers are the names of the cities (*n* = number of people living with HIV/AIDS). (1) Abaetetuba (*n* = 2), (2) Acará (*n* = 2), (3) Altamira (*n* = 1), (4) Ananindeua (*n* = 53), (5) Baião (*n* = 1), (6) Barcarena (*n* = 3), (7) Belém (*n* = 159), (8) Benevides (*n* = 14), (9) Breves (*n* = 2), (10) Bujaru (*n* = 1), (11) Cachoeira do Arari (*n* = 3), (12) Cametá (*n* = 2), (13) Capanema (*n* = 1), (14) Capitão Poço (*n* = 2), (15) Castanhal (*n* = 7), (16) Colares (*n* = 1), (17) Concórdia do Pará (*n* = 2), (18) Curuçá (*n* = 1), (19) Garrafão do Norte (*n* = 1), (20) Gurupá (*n* = 2), (21) Igarapé-Miri (*n* = 4), (22) Ipixuna do Pará (*n* = 1), (23) Irituia (*n* = 1), (24) Joanes (*n* = 1), (25) Limoeiro do Ajuru (*n* = 1), (26) Mãe do Rio (*n* = 2), (27) Marabá (*n* = 2), (28) Marapanim (*n* = 2), (29) Marituba (*n* = 17), (30) Mocajuba (*n* = 1), (31) Moju (*n* = 4), (32) Muaná (*n* = 1), (33) Nova Timboteua (*n* = 2), (34) Novo Repartimento (*n* = 1), (35) Oeiras do Pará (*n* = 1), (36) Ourém (*n* = 1), (37) Ourilândia do Norte (*n* = 1), (38) Pacajá (*n* = 1), (39) Paragominas (*n* = 5), (40) Ponta de Pedras (*n* = 3), (41) Portel (*n* = 2), (42) Salinópolis (*n* = 2), (43) Salvaterra (*n* = 3), (44) Santa Bárbara (*n* = 4), (45) Santa Isabel do Pará (*n* = 7), (46) Santa Maria do Pará (*n* = 9), (47) Santarém Novo (*n* = 1), (48) Santo Antônio da Ponta (*n* = 1), (49) Santo Antônio do Tauá (*n* = 1), (50) São Caetano de Odivelas (*n* = 1), (51) São João de Pirabas (*n* = 2), (52) São Miguel do Guamá (*n* = 2), (53) São Sebastião da Boa Vista (*n* = 2), (54) Soure (*n* = 1), (55) Tailândia (*n* = 2), (56) Terra Alta (*n* = 1), (57) Tomé-Açu (*n* = 3), (58) Traquateua (*n* = 1), (59) Tucumã (*n* = 2), (60) Ulianópolis (*n* = 1), (61) Vigia (*n* = 5), (62) Viseu (*n* = 2), and (63) Xinguara (*n* = 1). Belém (8) is the state capital.

The mean age was 42.3 years (ranging from 18 to 69 years of age). The majority of PLWHA were male, aged between 31 and 50 years, heterosexual, unmarried, had an educational level of secondary school or higher, and were living in cities of the interior of the state. Routine condom use during sexual intercourse was reported by 58.7% and up to one sexual partner over last year for 85.9% of the participants. Sixty percent had no history of other sexually transmitted infection (STI). Table 1 summarizes the epidemiological characteristics of PLWHA and HTLV coinfection of this study. The geographical distribution of the cities with participants enrolled in this study is shown in Figure 1.

**TABLE 1:** Demographic characteristics of people living with HIV/AIDS and prevalence of HTLV coinfection in the State of Pará, Northern Brazil.

**Characteristics**	**HIV**		**HTLV/HIV**		***p-*value**	**OR (95% CI)**
	***N***	**%**	***N***	**%**		
**Age (years)**						
18–30	73	20.1	1	20.0	1.00^a^	–
>30	290	79.9	4	80.0		
**Sex**						
Male	202	55.2	1	20.0	0.18^a^	0.20 (0.02–1.80)
Female	161	44.8	4	80.0		
**Marital status**						
Unmarried	258	71.2	4	80.0	1.00^a^	1.63 (0.20–14.74)
Married	105	28.8	1	20.0		
**Monthly income**						
Up to one wage*	157	43.2	2	40.0	1.00^a^	0.87 (0.14–5.30)
More than one wage	206	56.8	3	60.0		
**Sexual orientation**						
Homosexual + Bisexual	107	29.3	1	20.0	1.00^a^	0.60 (0.07–5.41)
Heterosexual	256	70.7	4	80.0		
**Source**						
City of Belém	167	45.4	3	60.0	0.66^a^	1.81 (0.30–10.93)
Interior of Pará	201	54.6	2	40.0		
**Length of education**						
Up to primary school	115	31.8	2	40.0	0.99^a^	1.44 (0.24–8.72)
Secondary school or more	248	68.2	3	60.0		
**Condom use**						
Rarely + Sometimes	148	41.3	4	80.0	0.17^a^	5.81 (0.64–52.51)
Always	215	58.7	1	20.0		
**Number of sexual partners (last year)**						
0–1	311	84.7	5	100.0	0.78^b^	–
>1	52	14.3	0	0.0		
**STI History**						
Yes	146	40.2	3	60.0	0.40^a^	2.23 (0.37–13.51)
No	217	59.8	2	40.0		

*Average of the Brazilian minimum wage from 2005 to 2006= R$ 325,00 (equivalent to US$ 81,00).

*^*a*^Fisher Exact test.*

*^*b*^G test.*

*OR, odds ratio; 95% CI, 95% confidence interval.*

**FIGURE 2:**
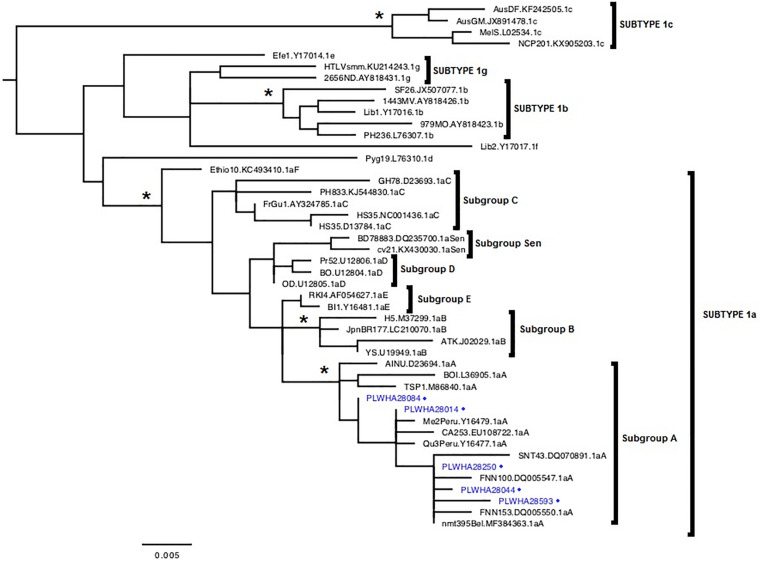
Rooted phylogenetic tree showing the evolutionary relationship of human T-lymphotropic virus-1 strains, including the strains detected in this study [indicated with people living with HIV/AIDS (PLWHA) + number + blue dot], and others obtained from the National Center for Biotechnology Information. The tree was constructed by the maximum-likelihood method after alignment of 548 nucleotides of the *5’* long terminal repeat (*LTR*) region. The branch support was calculated with the approximate likelihood-ratio (aLRT) SH-like test. *aLRT values ≥0.95.

In 368 PLWHA, five (1.4%) had anti-HTLV-1/anti-HTLV-2 antibodies using ELISA. Amplification of the albumin gene fragment was detected in the five PLWHA samples, indicating success in DNA isolation. HTLV-1 DNA was detected in all samples positive for anti-HTLV-1/HTLV-2 antibodies, which were all genotyped as belonging to the subtype 1a (Cosmopolitan), subgroup A (Transcontinental) (Figure 2). Table 2 shows the epidemiological information of PLWHA infected with HTLV-1. HTLV-2 DNA was not detected in PLWHA samples with positive results for anti-HTLV-1/HLTV-2 antibodies. All individuals who presented HIV/HTLV-1 coinfection were clinically asymptomatic during the study and were using antiretroviral therapy (ART).

**TABLE 2:** Epidemiological characteristics of people living with HIV/AIDS coinfected with HTLV-1 in Pará state, Amazon region of Brazil.

	**#1**	**#2**	**#3**	**#4**	**#5**
Age (years)	42	28	34	33	42
Gender	Female	Male	Female	Female	Female
Sexual orientation	Hetero	Homo	Hetero	Hetero	Hetero
Marital status	Single	Single	Married	Single	Single
Education	Secondary	Primary	Primary	Primary	Secondary
Resident in	Belém	Belém	Marituba	Ponta de Pedras	Belém
Occupation	Unemployed	Student	Maid	Maid	Cooker
Condom use	No	No	No	No	Yes
Sexual partners*	No	Single	Single	Single	Single
STI history	No	Yes	Yes	Yes	No

*Sexual partners in the last year.

From the comparison of the numbers of positive and negative cases of HTLV infections of this study with findings from previous studies, a decreasing trend of cases of HTLV infections in PLWHA was detected (*A* = -13.02; χ^2^ = 11.26; *p* < 0.05). Similarly, a decreasing trend in cases of HTLV-2 infection has also been identified (*A* = −4.00; χ^2^ = 4.46; *p* = 0.03).

## Discussion

To date, this study is the third epidemiological survey in the State of Pará over a period of 22 years that investigated the HIV/HTLV coinfection. A big shift has been observed over time, showing a continuous decrease in HIV/HTLV coinfection prevalence, dropping from 8% in 1998 (Vallinoto et al., 1998) to 3.5% in 2005 (Laurentino et al., 2005) and to 1.3% in the current investigation. Another important shift was the subtype prevalence; while the previous studies showed a higher prevalence of HTLV-2 (overall rate of 1.8:1), our current results showed the HTLV-1a as the exclusive subtype. In addition, this study distinguishes from the previous ones (Vallinoto et al., 1998; Laurentino et al., 2005) due to the inclusion of a higher number of cities and small towns (less than 50,000 inhabitants).

Worldwide, few studies have described the epidemiological characteristics of HIV/HTLV coinfection. After dropping from 8% in 1998 (Vallinoto et al., 1998) to the current 1.3% prevalence rate, the Pará state became similar to Mozambique (1.55%) (Augusto et al., 2017), lower than Nigeria (4.9%) (Nasir et al., 2015) but still higher than Sierra Leone, where no cases of HTLV coinfection was observed in PLWHA (Yendewa et al., 2019). In Brazil, this rate is relatively similar to the states of Piaú (1.61%) (de Oliveira et al., 2012), Pernambuco (1.5%) (Ribeiro et al., 2019), and Santa Catarina (1.1%) (Marcon et al., 2019) and just a bit above the State of Goiás (0.8%) (Kozlowski et al., 2016) and below Rio Grande do Sul (2.9%) (Galetto et al., 2014).

The Amazon region (Northern Brazil) is considered hyperendemic for HTLV-2, especially HTLV-2c, which has been described in Amerindian tribes from the 1990s to date (Ishak et al., 1995, 2001; Santos et al., 2009; Braço et al., 2019). Furthermore, HTLV-1 and HTLV-2 were detected in PLWHA in all Brazilian studies previously referenced (Vallinoto et al., 1998; Laurentino et al., 2005; Santos et al., 2009; de Oliveira et al., 2012; Sequeira et al., 2012; Galetto et al., 2014; Kozlowski et al., 2016), and now, no cases of HTLV-2 were identified in the current investigation. This strongly suggests a change in seroprevalence rate of HTLV-2 in PLWHA in the state. Interestingly, HTLV-2 subtype is still circulating locally, since it has been described in health blood donors (Santos et al., 2009), Afro-Brazilian quilombolas (Vallinoto et al., 2006), and pregnant women (Sequeira et al., 2012).

A remarkable characteristic found here is that the HTLV carriers showed no history of multiple sexual partners and illicit drugs use, which is a different profile from the previous findings in other Brazilian studies (Morimoto et al., 2005; Kozlowski et al., 2016; Marcon et al., 2019). However, these data also showed the inconsistent use of condoms during sexual intercourse for a significant portion of the participants (41.3%), especially because all were aware of their HIV seropositivity. It suggests a sexual risk behavior to acquire other STIs (including HTLV) and also the spread of HIV infection to partners. This scenario is different from that observed in the local study that took place in 1998 (Vallinoto et al., 1998), which showed that HIV/HTLV coinfection was found predominantly in injecting drug use and promiscuity.

The change in the epidemiological scenario observed in the present study, with the predominance of HTLV-1 instead of HTLV-2, may reflect a shift in the coinfection profile by HTLV in PLWHA in the State of Pará. The impact of this change has yet to be assessed, considering that all individuals participating in the present study were asymptomatic, similar to what was previously described in the city of Belém, Pará, where HTLV-2 predominated in this population (Vallinoto et al., 1998).

Our group is conducting clinical and laboratory monitoring of these individuals in order to better describe the impact of HIV-1/HTLV-1 coinfection in the clinical course of these patients in the future, bearing in mind that this coinfection could cause a greater risk of developing HAM, different from what has already been observed in HTLV-2 coinfection (Dhasmana and Taylor, 2014).

This study has limitations that should be considered. First, this epidemiological investigation selected a sample of PLWHA attending a reference unit for HIV/AIDS control and may not represent a full picture by excluding those who abandoned treatment. It is important to note that in Brazil, the public health system offers free universal treatment for individuals infected by the HIV. Another limitation is that qPCR tests were only carried out in individuals who tested positive to HTLV-1/2 screening using ELISA, so recent infections with a small concentration of anti-HTLV-1/2 antibodies may not have been detected, thus resulting in false-negative test result (Morais et al., 2017). The small number of HIV/HTLV coinfection individuals limits statistical analysis and the ability to identify factors associated with viral infections; thus, future studies with a larger sample size are necessary to research the real factors associated with HTLV infection in the PLWHA population in the State of Pará. Finally, the ability to establish causality is limited in a cross-sectional study.

This study identified that the prevalence of HIV/HTLV coinfection is consistently dropping over the last 22 years in the State of Pará when the first epidemiological survey took place. A shift of HTLV subtypes was also observed—from a predominance of HTLV-2 infection in the past to an exclusively HTLV-1a (Cosmopolitan subtype) in the current investigation. In addition, the presence of HTLV-2 was not detected in PLWHA, especially HTLV-2c, which is hyperendemic in several populations in Northern Brazil. There was no significant association between economic, sociodemographic, and behavioral characteristics in HIV/HTLV coinfection. These results suggest that the prevalence of HTLV infection in the PLWHA population in the State of Pará, North Region of Brazil, is lower than those previously related for the same region and that a shift regarding the HTLV subtype circling in PLWHA may also be occurring, with the predominance of HTLV-1a (Cosmopolitan subtype).

## Data Availability Statement

All datasets presented in this study are included in the article/supplementary material.

## Ethics Statement

The studies involving human participants were reviewed and approved by Human Research Ethics Committee of the Health Sciences Institute, Federal University of Pará. The patients/participants provided their written informed consent to participate in this study.

## Author Contributions

SA and LM contributed to the conceptualization. MS, CM, RF, and VA contributed to data curation. SA, AR, MB, RL, FF, and VA contributed to the investigation and methodology. SL, AO-F, and LM contributed to the formal analysis. SA, AO-F, and LM contributed to writing the original draft. SA, RL, AO-F, and LM contributed to writing, reviewing, and editing. LM contributed to the project administration. All authors contributed to the development of the research, read, and approved the final manuscript.

## Conflict of Interest

The authors declare that the research was conducted in the absence of any commercial or financial relationships that could be construed as a potential conflict of interest.

## Footnotes

1 https://www.ncbi.nlm.nih.gov

2 http://tree.bio.ed.ac.uk/software/figtree/

3 https://www.openepi.com/Menu/OE_Menu.htm
